# Adjunctive citrus flavonoids after endovenous microwave ablation for great saphenous vein varicosis: A retrospective cohort study

**DOI:** 10.1016/j.jvsv.2026.102586

**Published:** 2026-07-28

**Authors:** Yiqi Rui, Wenjun Chen

**Affiliations:** Department of General Surgery, Geriatric Hospital of Nanjing Medical University, Nanjing, Jiangsu, China

**Keywords:** Great saphenous vein varicosis, Citrus flavonoids, Endovenous microwave ablation, Venous hemodynamics, Retrospective cohort, Micronized purified flavonoid fraction

## Abstract

**Objective:**

To evaluate whether adjunctive citrus flavonoids are associated with improved venous hemodynamics and short-term outcomes after endovenous microwave ablation (EMA) for great saphenous vein varicosis in a retrospective cohort.

**Methods:**

This single-center retrospective cohort study included 254 patients with great saphenous vein varicosis treated between January 2022 and July 2025. Patients were grouped according to the actual treatment received: EMA alone (n = 126) or EMA plus micronized purified flavonoid fraction (MPFF; n = 128). In the combination group, MPFF was administered at 1000 mg twice daily (2000 mg/d) for 2 weeks; this was twice the usual approved dose for venolymphatic insufficiency. The primary outcome was overall clinical response at 2 weeks. Secondary outcomes included Venous Clinical Severity Score, Aberdeen Varicose Vein Questionnaire, Chronic Venous Insufficiency Questionnaire, venous hemodynamic parameters, exploratory endothelial biomarkers (such as endothelin-1, nitric oxide, and circulating endothelial cells), treatment-related adverse events, and 6-month ultrasound findings.

**Results:**

Compared with EMA alone, adjunctive MPFF was associated with a higher overall clinical response and greater improvements in Venous Clinical Severity Score, Aberdeen Varicose Vein Questionnaire, and Chronic Venous Insufficiency Questionnaire scores (all *P* < .05). Mean and peak venous flow velocities improved more in the combination group (*P* < .05). The combination group also showed lower endothelin-1 and circulating endothelial cell levels and higher nitric oxide levels after treatment (*P* < .05). Recorded adverse events did not differ significantly between groups (*P* > .05). At 6 months, the combination group showed a higher rate of complete occlusion and less residual reflux (*P* < .05).

**Conclusions:**

In this retrospective cohort, adjunctive citrus flavonoids after EMA were associated with better early symptom scores, venous hemodynamic indices, and endothelial biomarker profiles without a detected increase in recorded adverse events. As the study was observational and used a nonstandard MPFF dose, these findings should be interpreted as associations rather than evidence of causality, proof of safety, or support for the 2000 mg/d regimen. Prospective randomized trials using approved dosing are needed to confirm these findings.

**Article Highlights**

• **Type of Research:** Single-center retrospective cohort study
• **Key Findings:** Among 254 patients, endovenous microwave ablation (EMA) plus micronized purified flavonoid fraction was associated with a higher short-term clinical response and greater improvement in Venous Clinical Severity Score, Aberdeen Varicose Vein Questionnaire, and Chronic Venous Insufficiency Questionnaire scores than EMA alone, without a significant increase in recorded adverse events.
• **Take Home Message:** Micronized purified flavonoid fraction may be a useful postoperative adjunct to EMA for early symptom control, but causality, long-term efficacy, and effects on vein occlusion require prospective randomized confirmation.

The great saphenous vein (GSV), the longest superficial vein in the human body, originates from the medial aspect of the dorsal venous arch of the foot, passes anterior to the medial malleolus, ascends along the medial side of the leg and thigh, and ultimately drains into the femoral vein.^1^ GSV varicosis (GSVV) is among the most common conditions encountered in vascular surgery. Its global prevalence is estimated at approximately 20%, with incidence increasing steadily with age.^2^ The disease is primarily attributed to valvular incompetence and structural weakness of the venous wall, which lead to venous reflux and elevated venous pressure in the lower limbs. Clinically, patients present with tortuous and dilated superficial veins, limb heaviness, swelling, and discomfort; in advanced cases, venous ulcers may develop, substantially impairing quality of life.^3^^,^^4^

Current treatment options for GSVV include high ligation with vein stripping, endovenous laser ablation, radiofrequency ablation, endovenous microwave ablation (EMA), and sclerotherapy. Contemporary international guidelines support endovenous treatment for superficial truncal reflux when technically feasible, and the Society for Vascular Surgery/American Venous Forum/American Vein and Lymphatic Society guideline is endorsed by the International Union of Phlebology.^5^^,^^6^ Venoactive drugs, including micronized purified flavonoid fraction (MPFF), are recommended primarily for symptom relief in chronic venous disease rather than as substitutes for correction of venous reflux.^7^ Among thermal options, EMA induces thermal injury through microwave energy, resulting in collagen contraction, vein wall denaturation, and eventual occlusion. It is minimally invasive and allows for rapid recovery, which has led to its increasing use in recent years.^8^

However, postoperative pain and swelling can occur after all endovenous thermal ablation techniques as transient inflammatory responses and are not unique to EMA. Recanalization and recurrent reflux are generally medium- to long-term outcomes and are influenced largely by patient anatomy, reflux extent, vein diameter, and procedural technique. Therefore, adjunctive medical therapy should be evaluated mainly for early symptom control, venous hemodynamics, and recovery, whereas claims regarding long-term procedural durability should remain cautious.

Citrus flavonoids, bioactive compounds extracted from citrus fruits, have demonstrated anti-inflammatory, antioxidant, and antiplatelet properties.^9^ Han et al^10^ reported that citrus flavonoid tablets combined with endovenous intervention reduced venous severity scores and alleviated limb swelling in patients with deep vein thrombosis (DVT). However, evidence that citrus flavonoids reduce post-thrombotic syndrome remains insufficient, their follow-up period was relatively short, and whether these findings can be directly extrapolated to patients with GSVV undergoing EMA remains unclear. In the light of these considerations, the present study investigates the association of adjunctive citrus flavonoids with venous hemodynamics, exploratory endothelial biomarkers, and short-term clinical outcomes in patients with GSVV undergoing EMA, with the aim of informing optimization of minimally invasive treatment strategies.

## Methods

### Study population

A total of 254 patients with GSVV treated at the Geriatric Hospital of Nanjing Medical University between January 2022 and July 2025 were retrospectively reviewed. This study was designed as a single-center retrospective cohort study. The study was approved by the Institutional Ethics Committee, and the requirement for informed consent was waived because all data were anonymized before analysis.

Inclusion criteria were as follows: (1) diagnosis of GSVV according to established criteria,^11^ confirmed by color Doppler ultrasound study; (2) a GSV trunk diameter ≥5 mm with reflux of varying severity (reflux duration >1 s); (3) Clinical, Etiological, Anatomical, and Pathophysiological classification of C2-C5; (4) first-time treatment with EMA; (5) age between 18 and 80 years; and (6) complete clinical data with a follow-up duration of at least 6 months.

Exclusion criteria included: (1) concomitant DVT, arteriovenous fistula, or acute superficial thrombophlebitis; (2) severe arterial insufficiency (ankle-brachial index <0.5) or lower-extremity arteriosclerosis obliterans; (3) previous lower limb venous surgery or endovascular intervention; (4) allergy to citrus flavonoids or related excipients; (5) pregnancy or lactation; (6) severe cardiac, hepatic, or renal dysfunction, or malignancy; and (7) psychiatric or cognitive disorders that would preclude reliable assessment.

Treatment allocation was not randomized. Patients were classified according to the actual treatment received in routine clinical practice after clinician-patient discussion. The EMA group included 126 patients treated with EMA alone, whereas the combination group comprised 128 patients who received citrus flavonoids in addition to EMA. Baseline characteristics were comparable between groups (*P* > .05), as shown in Table I.

**Table I:** Baseline characteristics of the two groups [means ± SD, n (%)]

Target	Joint group (N = 128)	EMA group (N = 126)	*χ*^2^/*t*/*U* value	Parking lot value
Sex			0.130	0.718
Male	52 (40.63)	54 (42.86)	–	–
Female	76 (59.38)	72 (57.14)	–	–
Age, years	58.74 ± 9.23	57.16 ± 10.05	1.305	0.193
Body mass index, kg/m^2^	22.88 ± 2.67	23.42 ± 2.15	−1.774	0.077
Diameter of the main trunk of the great saphenous vein, mm	7.16 ± 1.04	6.98 ± 0.92	1.460	0.145
Course of a disease, years	5.32 ± 1.56	5.13 ± 1.87	0.880	0.380
Intravenous backflow time (small size)	2.97 ± 0.72	3.12 ± 0.78	−1.593	0.112
CEAP grading	–	–	0.185	0.853
Centigrade^2^ level	42 (32.81)	38 (30.16)	–	–
Centigrade^3^ level	49 (38.28)	52 (41.27)	–	–
Centigrade^4^ level	25 (19.53)	26 (20.63)	–	–
Centigrade^5^ level	12 (9.38)	10 (7.94)	–	–
Lateral separation of the affected limb			0.057	0.811
Left side	72 (56.25)	69 (54.76)	–	–
Right side	56 (43.75)	57 (45.24)	–	–
Complication				
Hypertension	36 (28.13)	45 (35.71)	1.684	0.194
Diabetes	19 (14.84)	28 (22.22)	2.292	0.130
Hyperlipidemia	31 (24.22)	22 (17.46)	1.756	0.185
Coronary heart disease	10 (7.81)	15 (11.90)	1.198	0.274

*CEAP*, Clinical, Etiological, Anatomical, and Pathophysiological classification; *EMA*, endovenous microwave ablation.

### Treatment protocol

EMA group: All procedures were performed using an EMA system (ECO-100A1, Nanjing Yigao Medical Technology Co, Ltd). Under ultrasound guidance and tumescent local anesthesia, a 0.5 cm skin incision was made 1 to 2 cm above the medial malleolus of the affected limb. The subcutaneous tissue was bluntly dissected to expose the trunk of the GSV. This distal cut-down approach was used as the institutional protocol during the study period to secure venous access in patients with tortuous distal refluxing GSV segments; ultrasound-guided percutaneous access is now generally preferred when feasible, and the potential influence of this access strategy is acknowledged in the limitations.

After successful venous puncture, a 6F introducer sheath was inserted, followed by placement of a microwave ablation catheter. With continuous ultrasound monitoring, the catheter tip was advanced to a position of 2 to 3 cm distal to the saphenofemoral junction, avoiding the valve. Ablation was initiated at a power setting of 50 W using a segmental pullback technique. The proximal trunk, including the first three 1-cm segments distal to the saphenofemoral junction, was treated for 12 sec/cm withdrawal. The remaining refluxing thigh GSV and refluxing calf GSV segments were treated at 50 W for 6 to 8 sec/cm withdrawal, adjusted according to venous diameter and real-time ultrasound response.

Ablation targeted the refluxing GSV trunk identified on preoperative duplex ultrasound findings. When reflux did not extend to the distal calf, ablation was stopped at the most distal refluxing segment rather than routinely ablating a normal ankle-level segment. This approach avoided routine distal ablation of normal ankle-level segments, which may increase the risk of saphenous nerve irritation and postoperative ankle edema.

During the procedure, real-time ultrasound guidance was used to monitor changes in venous wall echogenicity. The appearance of hyperechoic gas signals indicated adequate vein closure. Following the procedure, manual compression was applied to the puncture site for 5 to 10 minutes to achieve hemostasis. Once bleeding was excluded, the site was dressed with sterile gauze, and the limb was compressed using an elastic bandage. Compression therapy, early ambulation, hydration advice, and routine rehabilitation measures were identical between groups. Adjunctive phlebectomy or foam sclerotherapy was not planned as part of the index protocol in either group.

Combination group: In addition to the EMA procedure described above, patients received MPFF tablets (LES Laboratoires Servier; Approval No. HJ20140455; 500 mg/tablet, expressed as total flavonoids). Treatment was initiated on postoperative day 1 at a dose of 1000 mg twice daily (morning and evening) and continued for 2 weeks. The usual approved dose for venolymphatic insufficiency is 500 mg twice daily (1000 mg/d); therefore, the regimen documented in this cohort was twice the recommended daily dose. This was a clinician-selected, nonstandard postoperative regimen used in routine local practice, rather than a dose assigned prospectively by the investigators. We retained the actual recorded exposure to avoid misclassification, but we do not endorse this regimen. As the study included no standard-dose MPFF comparison group, it cannot establish the efficacy, safety, or appropriateness of 2000 mg/d, and the findings should not be used to justify dose escalation.

### Outcome measures

(1) Clinical efficacy: The primary outcome was overall clinical response at 2 weeks, evaluated according to previously published criteria.^12^ Outcomes were classified as follows: markedly effective, defined as near-complete resolution of lower limb symptoms (such as heaviness, aching, and edema) and varicosities with no reflux detected in the treated GSV segment on color Doppler ultrasound study; effective, defined as significant symptomatic improvement with a clear reduction in the extent and severity of varicosities but residual reflux on ultrasound guidance; and ineffective, defined as no meaningful improvement in symptoms, persistent or recurrent varicosities, and ongoing reflux on imaging. The overall response rate was calculated as [(markedly effective + effective cases)/total cases × 100%].
(2) Clinical symptom scores: Disease severity and quality of life were assessed before treatment and at 2 weeks after treatment using the Venous Clinical Severity Score (VCSS), Aberdeen Varicose Vein Questionnaire (AVVQ), and Chronic Venous Insufficiency Questionnaire (CIVIQ). These tools were used because they capture complementary domains: VCSS reflects clinician-assessed venous disease severity, AVVQ assesses varicose vein-specific quality of life, and CIVIQ assesses broader chronic venous insufficiency-related quality of life. The VCSS includes 10 items scored from 0 to 3, yielding a total score of 0 to 30, with higher scores indicating more severe clinical disease. The AVVQ consists of 13 items converted to a 0 to 100 scale, with higher scores reflecting a greater negative impact on the quality of life. The CIVIQ comprises 20 items across four domains, giving a total score of 20 to 100; higher scores indicate better quality of life.
(3) Venous hemodynamic parameters: Venous hemodynamics were assessed using color Doppler ultrasound study; tissue perfusion was not directly measured. Peak velocity and mean flow velocity were assessed in a predefined patent proximal venous outflow segment adjacent to the saphenofemoral junction. The treated GSV trunk was not used for postoperative velocity measurements because successful ablation should result in luminal occlusion. Measurements were performed before treatment and at 2 weeks after treatment by experienced ultrasound physicians using the same institutional protocol.
(4) Endothelial function: Endothelial biomarkers were evaluated as exploratory secondary outcomes to reflect systemic endothelial activation associated with chronic venous hypertension and postoperative inflammatory stress. Biomarkers included endothelin-1 (ET-1; enzyme-linked immunosorbent assay), nitric oxide (NO; nitrate reductase method), and circulating endothelial cells (CEC; immunomagnetic bead separation combined with flow cytometry). Fasting peripheral venous blood (5 mL) was collected before treatment and at 2 weeks after treatment, centrifuged at 3000 rpm (radius 12 cm) for 10 minutes, and the serum was stored at −80 °C until analysis. In this retrospective analysis, only patients with complete paired biomarker data were included; all 254 included patients met this criterion, and biomarker testing was covered by the institutional ethics approval.
(5) Adverse events: Both MPFF-related and EMA-related adverse events during treatment and follow-up were recorded and compared between groups. Events included rash, headache, nausea and vomiting, diarrhea, postoperative pain, ecchymosis or hematoma, skin burn, wound infection, paresthesia or numbness, superficial thrombophlebitis, endothermal heat-induced thrombosis, and DVT.
(6) Prognosis: At the 6-month follow-up, vein occlusion and reflux were assessed using color Doppler ultrasound study. Occlusion of the treated GSV segment was classified as complete, partial, or absent based on luminal closure. Venous reflux was evaluated using the Valsalva maneuver in combination with ultrasound. Reflux duration was categorized as none (grade 0), <1, or >1 s.

### Statistical analysis

All analyses were performed using SPSS version 25.0 (IBM Corp.). Categorical variables are presented as n (%) and were compared using the *χ*^2^ test or Fisher exact test, as appropriate. Ordinal data were analyzed using the rank-sum test. Continuous variables are expressed as means ± standard deviation when normally distributed. Within-group pre- and post-treatment comparisons were assessed using paired-samples *t*-tests, whereas between-group comparisons of post-treatment values or changes from baseline were assessed using independent-samples *t*-tests. Where data were available, effect estimates were reported with 95% confidence intervals. No a priori sample-size or power calculation was performed because of the retrospective design; all eligible patients during the study period were included. Multivariable regression and propensity-score matching were not performed because the available retrospective dataset did not contain sufficient covariates for robust adjustment. Multiple secondary comparisons were exploratory and were not adjusted for multiplicity; therefore, *P* values for secondary outcomes should be interpreted cautiously. A two-sided *P* value <.05 was considered statistically significant.

## Results

### Clinical efficacy

The overall clinical response rate at 2 weeks was higher in the combination group than in the EMA group, and the difference reached statistical significance (*P* < .05). Detailed results are presented in Table II.

**Table II:** Comparison of clinical efficacy between the two groups, n (%)

Groups	N	Show an effect	Effective	Be invalid	Always have efficiency
United group	128	81 (63.28)	43 (33.59)	Four (3.13)	124 (96.88)
EMA group	126	60 (47.62)	51 (40.48)	15 (11.90)	111 (88.10)
*χ*^2^ value	–	–	–	–	7.072
Parking lot value	–	–	–	–	0.008

*EMA*, Endovenous microwave ablation.

### VCSS, AVVQ, and CIVIQ scores

All three scores improved after treatment in both groups. Compared with EMA alone, adjunctive MPFF was associated with greater improvement, reflected by lower VCSS and AVVQ scores and a higher CIVIQ score after treatment (*P* < .05).

**Fig 1:**
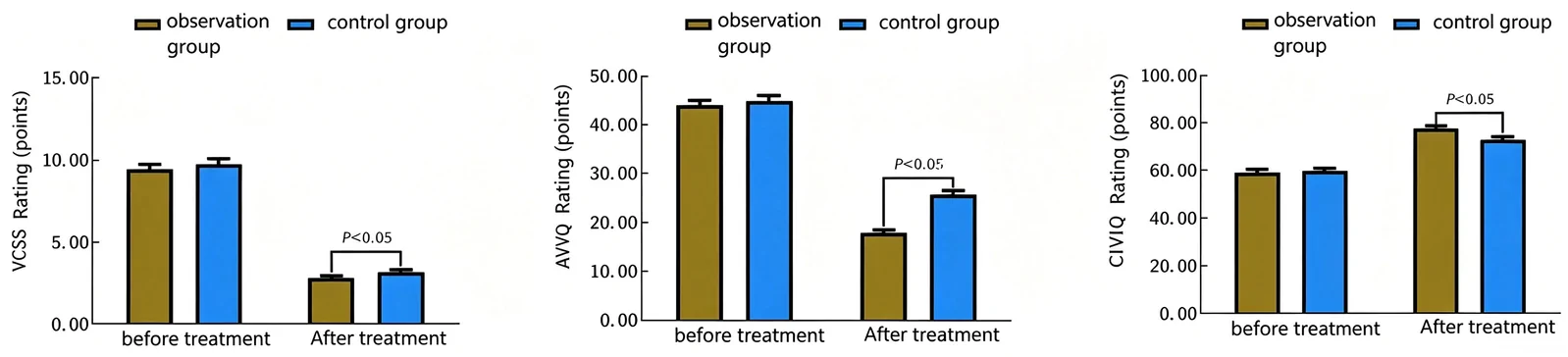
Comparison of Venous Clinical Severity Score (*VCSS*), Aberdeen Varicose Vein Questionnaire (*AVVQ*), and Chronic Venous Insufficiency Questionnaire (*CIVIQ*) scores between the two groups.

Results are summarized in Table III and illustrated in Fig 1.

**Table III:** Comparison of Venous Clinical Severity Score (*VCSS*), Aberdeen Varicose Vein Questionnaire (*AVVQ*), and Chronic Venous Insufficiency Questionnaire (*CIVIQ*) scores between the two groups (±s, points)

Groups	N	VCSS		AVVQ		CIVIQ	
		Before treatment	After treatment	Before treatment	After treatment	Before treatment	After treatment
United group	128	9.50 ± 1.72	2.87 ± 0.63^a^	44.26 ± 5.10	18.21 ± 3.12^a^	59.29 ± 6.10	78.44 ± 5.26^a^
EMA group	126	9.86 ± 1.93	3.24 ± 0.71^a^	45.10 ± 5.69	26.33 ± 4.11^a^	60.05 ± 5.78	73.21 ± 6.28^a^
*t* value		−1.570	−4.395	−1.239	−17.752	−1.019	7.200
Parking lot value		0.118	<0.001	0.216	<0.001	0.309	<0.001

*EMA*, Endovenous microwave ablation.

a *P* < .05 vs pretreatment within the same group.

### Hemodynamic parameters

Venous hemodynamic parameters increased after treatment in both groups. The increases in mean flow velocity and peak flow velocity in the predefined patent venous outflow segment were more pronounced in the combination group, with statistically significant differences compared with the EMA group (*P* < .05). Details are shown in Table IV.

**Table IV:** Comparison of hemodynamic parameters between the two groups (±s, cm/s)

Groups	N	Blood flow rate		Blood flow peak velocity	
		Before treatment	After treatment	Before treatment	After treatment
United group	128	13.75 ± 2.89	20.69 ± 3.18^a^	15.22 ± 3.10	22.18 ± 3.29^a^
EMA group	126	14.11 ± 3.26	18.10 ± 2.91^a^	15.81 ± 2.76	18.79 ± 3.12^a^
*t* value		−0.932	6.769	−1.601	8.424
Parking lot value		0.352	<0.001	0.111	<0.001

*EMA*, Endovenous microwave ablation.

a *P* < .05 vs pretreatment within the same group.

### Endothelial function

**Fig 2:**
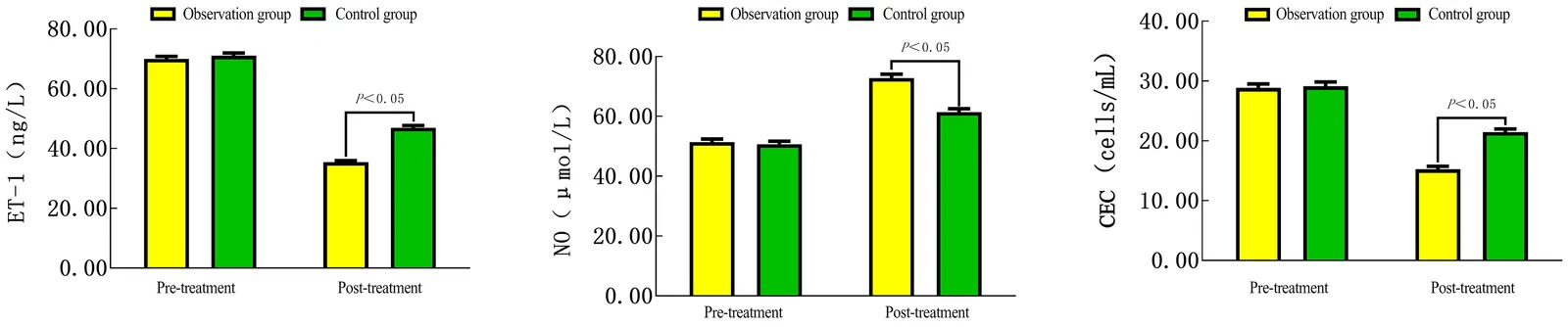
Comparison of endothelial function markers between the two groups. *CEC*, Circulating endothelial cells; *ET-1*, endothelin-1; *NO*, nitric oxide.

Exploratory endothelial biomarker profiles improved in both groups following treatment. Compared with the EMA group, the combination group showed lower levels of ET-1 and CEC alongside higher NO levels (*P* < .05). These findings describe systemic biomarker associations and do not by themselves prove direct endothelial protection. Results are presented in Table V and Fig 2.

**Table V:** Comparison of endothelial function markers between the two groups (±s)

Groups	N	ET-1, ng/L		NO, μmol/L		CEC, piece/mL	
		Before treatment	After treatment	Before treatment	After treatment	Before treatment	After treatment
United group	128	69.95 ± 4.87	35.40 ± 3.21^a^	51.36 ± 6.10	72.85 ± 7.02^a^	28.85 ± 3.74	15.26 ± 2.89^a^
EMA group	126	71.02 ± 5.26	46.98 ± 4.03^a^	50.71 ± 5.64	61.44 ± 6.21^a^	29.11 ± 4.16	21.44 ± 3.15^a^
*t* value		−1.683	−25.351	0.881	13.712	−0.524	−16.297
Parking lot value		0.094	<0.001	0.379	<0.001	0.601	<0.001

*CEC*, Circulating endothelial cells; *ET-1*, endothelin-1; *EMA*, endovenous microwave ablation; *NO*, nitric oxide.

a *P* < .05 vs pretreatment within the same group.

### Adverse events

Both MPFF-related and EMA-related adverse events were recorded, including gastrointestinal symptoms, rash, headache, postoperative pain, ecchymosis or hematoma, skin burn, wound infection, paresthesia or numbness, superficial thrombophlebitis, endothermal heat-induced thrombosis, and DVT. The overall incidence of recorded adverse events did not differ significantly between groups (*P* > .05). Detailed data are provided in Table VI.

**Table VI:** Comparison of adverse events between the two groups, n (%)

Groups	N	Rash	Have a headache	Nausea and vomiting	Diarrhea	Total incidence
United group	128	3 (2.34)	4 (3.13)	5 (3.91)	2 (1.56)	14 (10.94)
EMA group	126	1 (0.79)	2 (1.59)	4 (3.17)	3 (2.38)	10 (7.94)
*χ*^2^ value	–	–	–	–	–	0.668
Parking lot value	–	–	–	–	–	0.414

*EMA*, Endovenous microwave ablation.

### Prognosis

At the 6-month follow-up, the combination group demonstrated a higher rate of vein occlusion and a lower degree of venous reflux compared with the EMA group (*P* < .05). As occlusion and reflux are strongly influenced by procedural technique, these observational findings should be interpreted cautiously. The relevant findings are summarized in Table VII.

**Table VII:** Comparison of prognostic outcomes between the two groups, n (%)

Groups	N	Vascular occlusion			Reflow situation		
		Completely occluded	Partial occlusion	Not closed	Zero	<1	>1
United group	128	83 (64.84)	43 (33.59)	2 (1.56)	71 (55.47)	53 (41.41)	4 (3.13)
EMA group	126	70 (55.56)	41 (32.54)	15 (11.90)	58 (46.03)	47 (37.30)	21 (16.67)
*U* value		2.038			2.326		
Parking lot value		0.042			0.020		

*EMA*, Endovenous microwave ablation.

## Discussion

EMA has become an established treatment for GSVV. It works by delivering thermal energy that induces immediate collagen contraction and denaturation within the vein wall, ultimately leading to fibrotic occlusion of the lumen. This approach is minimally invasive and allows for rapid recovery, consistent with contemporary guideline support for endovenous treatment of superficial truncal reflux when technically feasible.^13^^,^^14^

Postoperative discomfort, pain, and swelling may occur after all endovenous thermal ablation techniques and usually reflect transient local inflammatory responses rather than a limitation unique to EMA. Thermal injury to the vein wall may also trigger local sterile inflammation and a transient hypercoagulable state, while reduced postoperative limb activity can slow venous return.^15^^,^^16^ Di Gangi et al^17^ reported thrombotic events after sclerotherapy and endovenous laser ablation in an observational cohort; however, this estimate should be interpreted cautiously because surveillance intensity and definitions may include symptomatic DVT, asymptomatic thrombosis, or endothermal heat-induced thrombosis. These considerations support careful postoperative monitoring and evaluation of adjunctive strategies for symptom control, but they do not establish that adjunctive medication can prevent thrombotic events or improve procedural durability.

Previous studies have explored combination approaches. Yang et al^18^ demonstrated that Danshen injection combined with surgery reduced postoperative complications and improved CIVIQ scores in patients with GSVV. Similarly, Cui et al^19^ reported that Maitong Ning decoction combined with EMA reduced whole blood viscosity, improved hemodynamics, and lowered the incidence of postoperative DVT. Jin et al^20^ also found that Xuefu Zhuyu decoction accelerated symptom relief and achieved favorable clinical outcomes when used alongside surgery.

The findings of the present study are consistent with these reports in showing better early symptom scores and quality of life measures in the combination group. However, as the present study was retrospective and nonrandomized, these results should be interpreted as associations between adjunctive MPFF use and postoperative outcomes rather than proof that MPFF caused the observed improvements.

The underlying mechanisms may be multifactorial. Although EMA effectively occludes the diseased vein, thermal injury may transiently aggravate local inflammation and edema. MPFF, derived from citrus sources, consists primarily of diosmin (90%) and hesperidin (10%). MPFF and related venoactive drugs are used mainly to relieve symptoms of chronic venous disease rather than to replace definitive correction of venous reflux.^5^^,^^7^ Diosmin has documented venoactive properties, including increased venous tone, improved lymphatic drainage, and enhanced hemodynamics.

Hesperidin, a natural flavonoid, exerts potent antioxidant effects. It scavenges free radicals, reduces oxidative stress-induced endothelial injury, and helps preserve vascular barrier integrity.^21^^,^^22^ In addition, hesperidin improves capillary resistance and microcirculatory function, which may further alleviate lower limb edema and discomfort. Acting in combination, these components provide complementary benefits that contribute to symptom relief and functional recovery.

In patients with GSVV, valvular incompetence leads to impaired venous return. Over time, this results in endothelial dysfunction and contributes to symptoms such as limb edema.^23^^,^^24^ CEC, which are shed into the bloodstream, are considered a direct marker of endothelial injury. ET-1 is a potent vasoconstrictor produced by endothelial cells and is associated with endothelial damage, whereas NO is a key vasodilator that helps maintain vascular tone and protects endothelial function.^25^, ^26^, ^27^

In the present study, the combination group showed higher venous flow velocity indices and NO levels, alongside lower ET-1 and CEC levels compared with the EMA group. These findings suggest an association between adjunctive MPFF and more favorable venous hemodynamic and systemic endothelial biomarker profiles. Huwait and Mobashir^28^ reported that diosmin, a flavonoid derived from citrus fruits, has anti-inflammatory and antioxidant properties and is widely used in the management of varicose veins, leg ulcers, and chronic venous insufficiency. A recent domestic review also highlighted the antioxidant and anti-inflammatory effects of hesperidin.^29^

Taken together, it is biologically plausible that diosmin and hesperidin act synergistically through anti-inflammatory and antioxidant pathways. Nevertheless, the present biomarker findings are exploratory. ET-1, NO, and CEC are systemic markers, and measurement after treatment of a single superficial vein cannot establish a direct local endothelial-protective effect. Further multicenter, large-scale randomized controlled trials, supported by mechanistic studies, are needed to clarify the underlying targets and pathways involved.

The incidence of recorded adverse events did not differ significantly between groups; however, this retrospective comparison was not designed or powered to establish the safety of the nonstandard 2000 mg/d MPFF regimen. At the 6-month follow-up, the combination group demonstrated a higher rate of complete vein occlusion and a lower degree of reflux. As vein occlusion and residual reflux are primarily determined by ablation technique, vein diameter, reflux extent, and follow-up assessment, these findings should be considered hypothesis-generating associations rather than evidence that MPFF improves procedural efficacy.

The dose requires specific caution. The usual approved MPFF dose for venolymphatic insufficiency is 1000 mg/d, whereas patients in the combination group received 2000 mg/d for 2 weeks. The absence of a statistically significant difference in recorded adverse events does not demonstrate safety, particularly because the study was retrospective and was not designed as a dose-safety study. We therefore do not recommend the 2000 mg/d regimen, and the observed associations should not be used to justify dose escalation.

In summary, adjunctive treatment with citrus flavonoids was associated with better early symptom relief, venous hemodynamic indices, and exploratory endothelial biomarker profiles in patients with GSVV undergoing EMA. However, the nonstandard 2000 mg/d dose and retrospective design prevent any conclusion regarding the safety or appropriateness of this regimen. Causal effects on endothelial function, reflux, and vein occlusion cannot be inferred from this observational study.

Several limitations should be acknowledged. First, this was a single-center retrospective cohort study, and the absence of randomization introduced potential selection bias and confounding by indication. Second, no multivariable adjustment or propensity-score matching was performed, and multiple secondary comparisons were not adjusted for multiplicity. Third, the MPFF regimen (2000 mg/d for 2 weeks) was twice the usual approved dose for venolymphatic insufficiency. This nonstandard exposure is a major limitation, restricts external validity, and prevents any recommendation regarding this dose; the absence of a statistical safety signal should not be interpreted as proof of safety. Fourth, postoperative management, medication adherence, distal access strategy, and extent of ablation may have influenced pain, edema, vein occlusion, and reflux. Fifth, the systemic endothelial biomarkers were exploratory, and the retrospective nature of their collection limits mechanistic interpretation. Finally, follow-up was limited to 6 months and the single-center population may limit generalizability. Prospective randomized controlled studies should use the approved MPFF dose (1000 mg/d), standardized ablation protocols, complete reporting of effect estimates and confidence intervals, adjusted analyses, and longer follow-up.

## Author Contributions

Conception and design: YR, WC

Analysis and interpretation: YR, WC

Data collection: YR

Writing the article: YR

Critical revision of the article: YR, WC

Final approval of the article: YR, WC

Statistical analysis: Not applicable

Obtained funding: Not applicable

Overall responsibility: WC

## Funding

None.

## Data availability

All data generated or analyzed during this study are included in this article. Further inquiries can be directed to the corresponding author.

## Ethics approval and consent to participate

This study was conducted in accordance with the Declaration of Helsinki on Human Research Ethics standards and was approved by the Institutional Review Board of Geriatric Hospital of Nanjing Medical University. The requirement for informed consent was waived because of the retrospective nature of the study, and all data were anonymized or maintained confidentially before analysis.
